# Emerging Glycolysis Targeting and Drug Discovery from Chinese Medicine in Cancer Therapy

**DOI:** 10.1155/2012/873175

**Published:** 2012-07-12

**Authors:** Zhiyu Wang, Neng Wang, Jianping Chen, Jiangang Shen

**Affiliations:** School of Chinese Medicine, The University of Hong Kong, Estates Building, 10 Sassoon Road, Hong Kong

## Abstract

Molecular-targeted therapy has been developed for cancer chemoprevention and treatment. Cancer cells have different metabolic properties from normal cells. Normal cells mostly rely upon the process of mitochondrial oxidative phosphorylation to produce energy whereas cancer cells have developed an altered metabolism that allows them to sustain higher proliferation rates. Cancer cells could predominantly produce energy by glycolysis even in the presence of oxygen. This alternative metabolic characteristic is known as the “Warburg Effect.” Although the exact mechanisms underlying the Warburg effect are unclear, recent progress indicates that glycolytic pathway of cancer cells could be a critical target for drug discovery. With a long history in cancer treatment, traditional Chinese medicine (TCM) is recognized as a valuable source for seeking bioactive anticancer compounds. A great progress has been made to identify active compounds from herbal medicine targeting on glycolysis for cancer treatment. Herein, we provide an overall picture of the current understanding of the molecular targets in the cancer glycolytic pathway and reviewed active compounds from Chinese herbal medicine with the potentials to inhibit the metabolic targets for cancer treatment. Combination of TCM with conventional therapies will provide an attractive strategy for improving clinical outcome in cancer treatment.

## 1. Introduction

Cancer is the second leading cause of mortality in human diseases worldwide. According to a national statistic report on the incidence and mortality in the USA, there were a total of 1,529,560 new cancer cases and 569,490 deaths from cancer occurring in 2010 [1]. Surgery, chemotherapy, and radiotherapy, either alone or in combination, have been considered as conventional strategies for cancer treatment in the last century. With the rapid development of molecular medicine, novel therapeutic approaches, such as immunotherapy, molecular targeted therapy, and hormonal therapy, have been proposed to improve clinical outcomes for cancer patients [2–4]. However, those therapeutic approaches are not always effective and clinical outcome in survival rates is still poor. One of the major problems is that cancer cells gradually develop resistances to those therapies. Seeking for new therapeutic approaches to improve outcome of cancer treatment is timely important.

Complementary and alternative medicine (CAM) attracts much attention for drug discovery in current cancer research [5, 6]. Among CAM modalities, TCM is particularly appreciated in both rural and well-developed urban areas of China based on its 5000-year-old history and a well-established theoretical approach [7, 8]. In China, Chinese herbal medicine is widely used as an adjunct therapy to reduce the resistances and side effects of cancer cells to chemotherapy and radiotherapy. Chinese herbal medicine in combination with chemotherapy and radiotherapy potentially improves clinical outcome in cancer treatment [9]. However, the relatively poor designs in many clinical reports, such as lack of quality standardization of herbal products, shortage of well-designed randomized controlled trials (RCT), and the limited sample size, bring difficulty to evaluate the benefits or disadvantages of herbal medicine for cancer treatment [10, 11]. TCM approaches have always been met with much skepticism and pessimism by the West. In fact, medicinal herbs are very important resources for drug discovery in cancer treatment. The direct experience from TCM on human subjects and its long history provide important cues for drug development. Of the 121 prescription drugs in use today for cancer treatment, 90 are originally derived from medicinal plants. Almost 74% of those drugs were discovered from folk medicine [12, 13]. In a previous review article, 48 out of 65 new drugs approved for cancer treatment during 1981–2002 were natural products, leading from natural products, or mimicked natural products in one form or another [14]. Among the drugs, the most well-known examples include *Vinca* alkaloids (vincristine, vinblastine, vindesine, vinorelbine), taxanes (paclitaxel, docetaxel), podophyllotoxin and its derivative (etoposide, teniposide), camptothecin and its derivatives (topothecan, irinothecan), anthracyclines (doxorubicin, daunorubicin, epirubicin, idarubicin), and others [15]. There is an impressive revival of seeking natural lead compounds for the generation of semisynthetic derivatives. Current progress in this aspect not only provide a chemical bank from natural sources for drug discovery but also bring better understanding for the chemical basis of Chinese herbal medicine for cancer treatment.

With its multiple components, TCM formulas and therapies are generally considered to regulate multiple cellular signal pathways [16]. Herbal medicine and their active components are promising sources for the designs of more effective and less toxic agents in cancer chemoprevention and treatment [17]. Many TCM products or single active components have been reported to inhibit a variety of processes in cancer cell growth, invasion, and metastasis by modulating a wide range of molecular targets, including cyclooxygenase-2 (COX-2), nuclear factor kappa B (NF-*κ*B), and nuclear factor erythroid 2-related factor 2 (Nrf2)-mediated antioxidant signaling pathways. A previous review article summarized the therapeutic targets of traditional Ayurvedic medicine for inflammation and cancer. The targets include growth factor signaling (e.g., epidermal growth factor); prostaglandin (e.g., COX-2); inflammation factors (e.g., inflammatory cytokines: TNF, IL-1, IL-6, chemokines); drug resistance genes (e.g., multidrug resistance); cell cycle proteins (e.g., cyclin D1 and cyclin E); angiogenesis factors (e.g., vascular endothelial growth factor); invasion mediators (e.g., matrix metalloproteinases); apoptosis related genes (e.g., bcl-2, bcl-X(L), XIAP, survivin, FLIP); proliferation factors (e.g., c-myc, AP-1, growth factors), [18]. With the development of systematic biology and bioinformatics, more attention has been paid to the synergistic effects of herbal medicine on “common” signal pathways involved in the proliferation, invasion, metastasis, and apoptosis of cancer cells. It is interesting to ask the question whether herbal medicine and it's derivatives can specially target on tumor biomarkers and affect survival of cancer cells.

Molecular targeted therapy has been attracted much attention in cancer treatment [19]. Ideally, the identified targets should be preferentially expressed or activated in cancer cells but not in normal cells. Combining molecular and genetic technologies, a number of small molecular inhibitors and antibodies targeting on kinases or oncogenes has been designed and synthesized [20, 21]. The most well-known examples include the small molecule Gleevec (targeting on BCR-ABL translocation associated with chronic myelogenous leukemia), and antibody-based molecule Herceptin (c-erbB-2 overexpression related to breast cancer), [22, 23]. However, primary and secondary resistances to these targeted molecules severely reduce their therapeutic efficacy [24, 25]. Therefore, seeking more distinctive molecular targets and their corresponding drug candidates become important tasks for oncologists.

Cancer cells can be distinguished from normal cells in several hallmarks. One of hall marks is that cancer cells have a fundamentally different bioenergetic metabolism from that of nonneoplastic cells. In normal cells, energetic metabolism mostly relies upon the process of mitochondrial oxidative phosphorylation which consumes glucose and oxygen to produce energy. In contrast, cancer cells have developed an altered metabolism that allows them to sustain higher proliferation rates [26]. Cancer cells could predominantly produce energy by glycolysis followed by lactic acid fermentation, even in the presence of oxygen—this is known as the “Warburg Effect” [27, 28]. Cancer glycolysis is a critical step in carcinogenesis and oncogenic activation [29, 30]. Targeting on glycolysis becomes an attractive strategy in cancer diagnosis and treatment clinically [31]. The inhibitors targeting some key enzymes showed promising anticancer effects and have been approved for clinical trials [32]. Chinese herbal medicine could specifically target on the molecules in the metabolic pathways of cancer. Recent progress leads to the discoveries of many active compounds derived from Chinese herbs with the properties of inhibiting cancer cell glycolysis activity. Therefore, in the present paper, we intend to review current progress about TCM-derived phytochemicals which specifically target the key enzymes and proteins involved in cancer glycolysis.

## 2. Glycolytic Pathway as a Target for Cancer Therapy

**Figure 1:**
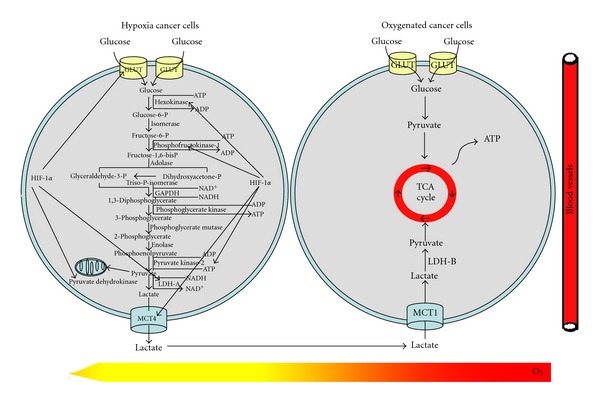
Glycolytic pathway and the role of HIF-1*α* in regulating glycolysis. Glucose was uptaken by increased expression of GLUT in hypoxia cancer cells. Through a series of enzyme reaction, glucose was finally metabolized into lactate and ATP, NAD^+^ was also regenerated by LDH-A for maintaining continuous glycolysis. Lactate was exhausted out of cancer cells by MCT4 and then uptaken by oxygenated cancer cells through MCT1. In the presence of oxygen, lactate is oxidized into pyruvate by LDH-B and pyruvate enters the tricarboxylic acid (TCA) cycle to produce ATP. HIF-1*α* was the main regulator of some enzymes expression in the glycolytic pathway, including GLUT-1, hexokinase, phosphofructokinase, pyruvate kinase, pyruvate dehydrogenase, LDH-A, and MCT.

Otto Heinrich Warburg, a pioneer in the study of respiration, made a striking discovery in the 1920s from extensive observation on the metabolic behavior of cancer cells. Even in the presence of oxygen, cancer cells prefer to metabolize glucose by glycolysis, a less efficient pathway for producing ATP [33]. The respiratory behavior was subsequently demonstrated in a various kinds of cancer cells and was called aerobic glycolysis [34–36]. The exact reasons why tumor cells exhibit elevated glycolysis and use this primitive and less energy-efficient pathway to generate ATP is still unclear. Accumulating evidences have suggested multiple mechanisms contributing to the unique phenomenon: (1) mitochondrial DNA mutations; (2) nuclear DNA mutations; (3) oncogenic transformation; (4) influences of the tumor microenvironment [37–39]. All these factors result in mitochondrial dysfunction and make cancer cells generate ATP much more dependently on the glycolytic pathway. Given the mitochondrial respiratory abnormality, cancer cells have to uptake much more glucose to produce enough ATP supporting rapid proliferation needs. At present, the phenomenon has been exploited clinically for the detection of tumors by fluorodeoxyglucose positron emission tomography (FDG-PET) [40]. Inhibition of aerobic glycolysis becomes an important strategy to preferentially kill cancer cells and to find anticancer agents based on Warburg hypothesis [41, 42]. As illustrated in Figure 1, cancer cells in the tumor mass could be divided into oxygenated and hypoxic cells. Hypoxic cancer cells predominately depend on glycolysis to produce energy. The glycolytic pathway is a series of metabolic reactions catalyzed by multiple enzymes or enzyme complexes. From the original glucose uptake to the final lactate production, the key steps include: (1) the increasing uptake of glucose by elevated expression of glucose transporter-1 (GLUT1) and sodium glucose cotransporter-1 (SGLT1); (2) active ATP generation reaction by upregulation of phosphoglycerate kinase (PGK) and pyruvate kinase (PK); (3) regeneration of NAD^+^ by lactate dehydrogenase (LDH); (4) out-transport and reuptake of lactate by monocarboxylate transporter (MCT), mainly MCT1 and MCT4 [43, 44]. In oxygenated cancer cells, the reuptaken lactate could be metabolized to pyruvate and reentered the mitochondrial tricarboxylic acid cycle to produce ATP. Each reaction in the glycolytic pathway is activated by a specific enzyme or enzyme complex. Interrupting any of the above proteins could lead to metabolism blockade followed by cell death. The activities of many enzymes in the pathway are controlled by two factors including c-myc and hypoxia inducible factor-1*α* (HIF-1*α*) [45, 46]. Many studies have demonstrated an increase in the activities of the glycolytic enzymes such as hexokinase, lactate dehydrogenase A (LDH-A), and glyceraldehydes-3-phosphate dehydrogenase (GAPDH) in various types of tumors and cancer cell lines [47–49]. In addition, silencing of these overexpressed enzymes, such as LDH-A or pyruvate kinase (PKM2), has been documented efficiency for inhibiting cancer cell proliferation, inducing apoptosis and reversing multidrug resistance [50–52]. Furthermore, some glycolytic enzymes are multifunctional proteins. For example, hexokinase and enolase play critical roles in transcription regulation [53, 54], while glucose-6-phosphate isomerase may affect cell motility [55]. Therefore, developing novel glycolytic inhibitors is an important direction in current cancer research. As TCM has held an important position in primary health care in China and been recently recognized by the West as a fertile source for revealing novel lead molecules for modern drug discovery, more and more herb-derived bioactive compounds have been identified for cancer therapy. Among them, several have been proved to be effective in suppressing cancer glycolytic activity by targeting on particular enzymes. Herein, we review current evidence on the studies of herbal medicine related to regulate several key enzymes in the glycolytic pathway including HIF-1*α*, hexokinase, and LDH-A.

## 3. Glycolytic Molecular Targets and Herb-derived Inhibitors

### 3.1. HIF-1*α*

**Figure 2:**
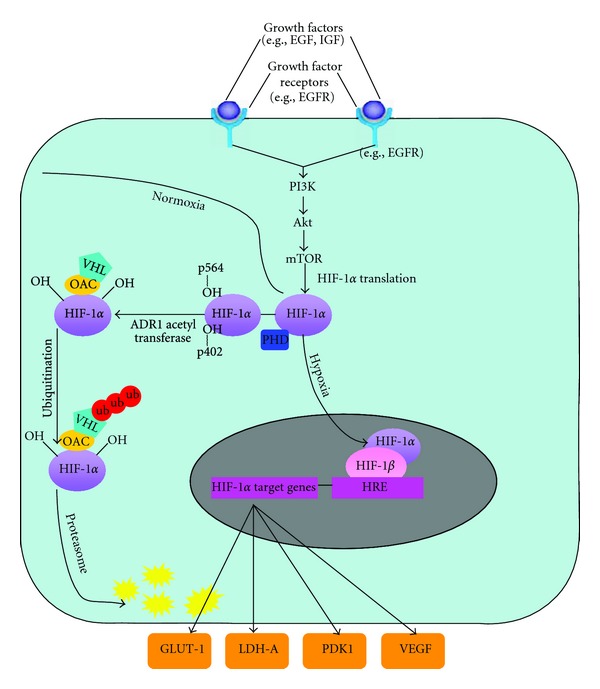
The intracellular HIF-1*α* regulation pathway in normoxia and hypoxia. Under normoxia, HIF-1*α* will be constituently ubiquitinated and subsequently degraded via proteasomal pathway after recruitment of von Hippel-Lindau protein (pVHL), which depends on the hydroxylation of proline residues on 564 and 402. However, under hypoxia, the praline hydroxylation of HIF-1*α* will be inhibited, HIF-1*α* will be translocated into the nucleus and combine with HIF-1*β*, then activate the transcription of a series of downstream genes including LDH-A, PDK1, GLUT-1, and VEGF. The levels of HIF-1*α* were also influenced by the PI3K/AKT pathway after stimulation of growth factors such as EGF and IGF.

HIF-1 is a basic helix-loop-helix heterodimeric transcriptional factor composed of *α* and *β* subunits [56]. HIF-1 is overexpressed in various types of cancer, and the levels of its activity have already been demonstrated closely to tumorigenicity, angiogenesis and also glycolytic activity [57, 58]. HIF-1*α* levels are primarily induced by hypoxia, growth factors, and oncogenes. As shown in Figure 2, under normoxia, HIF-1*α* is rapidly and continuously degraded by the ubiquitin-proteasome pathway. The prolyl hydroxylation of oxygen-dependent degradation domain (ODD) and binding with Von Hippel-Lindau (VHL) play a critical role in regulation of HIF-1*α* degradation. However, under hypoxic condition, the absence of oxygen prevents the prolyl hydroxylase process, allowing HIF-1*α* to accumulate and translocate to the nucleus, where it forms an active complex with HIF-1*β* and activates a series of downstream gene transcription [59, 60]. Besides hypoxia, the expression of HIF-1 was also regulated by other factors including oncogenes (p53, VHL, etc.), cytokines (EGF, TGF-*α*, IGF-1, and -2, etc.) and some posttranslational modifications including hydroxylation, ubiquitination, acetylation, and phosphorylation, [61, 62]. A number of glycolytic related genes are regulated by HIF-1*α*, such as GLUT-1, hexokinase, LDH-A, and PDK1. Interruption of HIF-1*α* signaling revealed to inhibit cancer growth in both *in vitro* and *in vivo* experimental models [63].

Recently, a remarkable progress has been made to seek selective HIF-1*α* inhibitors for cancer treatment from herbal medicine. Apigenin, a plant flavonoid compound, is considered as a typical HIF-1*α* inhibitor [64–72]. Apigenin is isolated from a traditional Chinese herb *Apiumgraveolensvar.dulce*. Apigenin has been shown to inhibit proliferation and induce apoptosis in a wide range of malignant cells including breast, ovarian, prostate, and lung cancer, [65–68]. Apigenin suppressed tumor angiogenesis by downregulating VEGF, a proangiogenic protein regulated by HIF-1*α* [69, 70]. Apigenin inactivated the PI3K/Akt pathway in prostate cancer cells [71, 72]. Apigenin reduced HIF-1*α* stability and HIF-1*α* mRNA expression in human prostate cancer PC3-M cells *via* PI3K/Akt/GSK-3*β* pathway [73]. Apigenin promoted HIF-1*α* degradation *via* disrupting HIF-1*α*-Hsp90 interaction under hypoxia [74]. Oral administration of apigenin resulted in tumor growth abrogation in prostate cancer xenografts, accompanied by inactivation of Akt, and induction of apoptosis [72]. Another study also revealed that apigenin *in vivo *administration significantly limited tumor growth and angiogenesis in both prostate and ovarian cancer models. Meanwhile, the expression of HIF-1*α* and VEGF were also down-regulated in apigenin-treated tumor samples [69]. Chrysin, isolated from *Oroxylum indicum (L.)Vent*, mediated apoptosis in various types of cancer including prostate, thyroid, and leukemia malignancies [75–77]. Chrysin significantly inhibited prostate cancer growth and angiogenesis with a decreased HIF-1*α* expression [78]. The suppression of HIF-1*α* expression could be related to different mechanisms including stimulation of PHD activity, disruption of HIF-1*α*-HSP90 interaction and direct inhibition of HIF-1*α* protein synthesis [78]. Epigallocatechin gallate (EGCG) is a widely spread flavonoid in Chinese herbs. The anticancer effects of EGCG were well documented [79, 80]. Many of its intracellular molecular targets, such as proteasomes, MAP kinases, VEGF, erythropoietin, and glucose transporters, are directly or indirectly regulated by HIF-1*α* [81, 82]. Several studies indicate that EGCG could inhibit HIF-1*α* expression by both blocking PI3K/Akt signaling pathway and reducing interaction between Hsp90 and HIF-1*α* [83]. Besides, curcumin, a well-validated anticancer compound extracted from *Curcuma longa*, has been found to interact directly with more than 30 different proteins including transcriptional factors (NF-*κ*B, AP-1, STAT, and *β*-catenin, etc.), growth factors and protein kinases (EGFR, ErbB-2, VEGF, EGF, MAPKs, and CXCR-4, etc.), inflammatory factors (TNF-*α*, IL-1*β*, IFN-*γ*, and COX-2, etc.), adhesion molecules (integrins, fibronectin, vitronectin, and collagen IV, etc.) and apoptosis-related proteins (death receptors, Bax, Bcl-2, and survivin, etc.) [84, 85]. Several studies also demonstrated that curcumin dose-dependently inhibited HIF-1*α* and HIF-1*β* gene at transcription level [86, 87]. Using luciferase reporter gene assay, an Indian herb *Ophiorrhiza trichocarpon* was identified to have the strongest HIF-1*α* inhibitive effects among more than 6,000 crude natural products. The extracts of *Ophiorrhiza trichocarpon *were shown to reduce hypoxia-induced HIF-1*α* accumulation to 22% relative to the normal control. Following bioactivity-guided fractionation assay validated camptothecin to be the best HIF-1*α* inhibitor among 84 fractions isolated from the medicinal plant [88]. Since the primary molecular target of camptothecin is established as human DNA topoisomerase I, its anticancer effects need to be further verified in animal experiments. Detail mechanisms in regulation of HIF-1*α* transcription activity remain to be further investigated. Terpenoids were also reported to inhibit HIF-1*α* activity. Nguyen et al. carried out screening assay for HIF-1*α* inhibitors from *Salvia miltiorrhiza* extracts by using luciferase gene reporting system. Diterpenes including sibiriquinone A, sibiriquinone B, cryptotanshinone, and dihydrotanshinone were finally identified to be strong HIF-1*α* inhibitors [89]. Although many herb-derived compounds are effective in suppressing HIF-1*α* activity, little compound is identified to specifically bind to HIF-1*α*. The structure-activity relationship and chemical optimization study are important topics for further studies on this direction. In addition, as many studies were conducted with cell systems,* in vivo *animal experiments are essentials to verify the bioactivities of targeting on HIF-1*α* activity contributing to their anticancer effects for drug development.

### 3.2. Hexokinase

**Figure 3:**
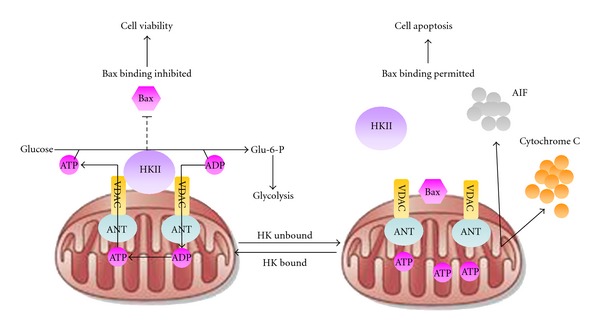
Antiapoptotic and metabolic roles for mitochondrial hexokinase II (HKII). (Left panel) Specific binding of HKII to the outer mitochondrial membrane (OMM) promotes ATP exchanges through complexes consisting of voltage-dependent anion channel (VDAC) and adenine nucleotide (ANT). The effluxed ATP could directly participate the transition from glucose to glucose-6-phosphate, which accelerates the glycolytic activity. Meanwhile, HKII binding to OMM also antagonizes Bax interaction with mitochondrial contact site, which prevents apoptosis occurrence; (Right panel) HKII unbound resulted in the “close” of VDAC-ANT channel, which induces Bax integration and potential changes between outer and inner mitochondrial membrane, and finally leading to release of cytochrome C and apoptosis-inducing factors (AIF) from mitochondrion.

Hexokinase (HK) controls the conversion of glucose to glucose-6-phosphate (G6P), which serves as the starting point for sugar to enter the glycolytic pathway or for glycogen synthesis [90]. Four isoforms of hexokinase have been identified in mammals, among which hexokinase II (HKII) is a major form responsible for maintaining the high glucose catabolic rates of malignant cells [91]. HKII overexpression was found in various types of cancers such as liver, breast, and lung cancers [92]. In addition to its glucose phosphorylation activity, HKII is capable of binding to the voltage-dependent anion channel (VDAC) on the mitochondrial outer membrane [93]. The specific binding not only allows efficient use of mitochondrial-generated ATP served as glycolytic fuel, but also stabilizes the mitochondrial membrane and prevents the release of pro-apoptotic factors, such as cytochrome C [94]. Disrupting the interaction between HKII and VDAC could inhibit cell proliferation and induce apoptosis through decreasing ATP supply and destabilization of mitochondrial membranes (Figure 3). Therefore, developing inhibitors targeting HKII is an interesting topic in anticancer drug development. 2-deoxyglucose (2-DG) and 3-bromopyruvate (3-BrpA) are well known HKII inhibitors [95, 96]. These pharmacological inhibitors have been proved to be effective in disrupting the binding of HKII to the mitochondrion, depleting ATP, inhibiting cell cycle progression, and inducing cell death. A TCM formula Ben Cao Xiao Ke Dan was revealed a strong inhibitory effect on HKII activity. However, exact phytochemicals in the formula accounting for this inhibitory effect remains unclear [97]. Methyl jasmonate, a plant lipid derivative, exists in many herbs and functions as a signaling molecule in the stress response. Methyl jasmonate was shown to induce apoptosis in various malignancies including prostate, cervical, and bladder cancers [98–100]. Recent studies found that its apoptosis-induction effects are closely correlated to the disruption of the interactions between HKII and VDAC [101]. Although some HKII inhibitors, such as 2-DG and 3-BrpA, were approved for clinical trials, the nonspecific inhibitions on all isoforms of HKs and normal cells might result in toxic effects when they are applied in patients. Therefore, to develop agents specifically targeting on HKII of cancer cells is a direction for further studies.

### 3.3. LDH-A

LDH-A is emerging as a novel therapeutic target in the glycolytic pathway. LDH has two subtypes: LDH-A, also called the skeletal muscle type or LDH-M, and LDH-B, also known as the heart type or LDH-H. LDH-A exhibits kinetic features suitable for conversion of pyruvate into lactate, whereas LDH-B has kinetic features suitable for conversion of lactate into pyruvate. LDH-A is an attractive target for cancer therapy because its expression is largely confined to skeletal muscle [102]. Moreover, human subjects with LDH-A deficiency show myoglobinuria under intense anaerobic exercise, and individuals with complete lack of LDH-A subunit have been documented with no apparent increase in hemolysis [103]. Numerous studies also demonstrated the overexpression of LDH-A in various types of cancer [104]. Considering the role of LDH-A in maintaining cancer cell energy metabolism, once its activity is inhibited, the energy-producing burden will be transferred to mitochondria, which may result in elevated oxidative stress and induce mitochondrial pathway apoptosis. Several studies have already found that the inhibition of LDH-A in cancer cells could stimulate mitochondrial respiration, decrease mitochondrial membrane potentials and finally lead to cancer cell death [105, 106]. Given LDH-A inhibition has no significant toxic effect on normal tissue, it is promising to develop novel LDH-A inhibitors. Gossypol is a polyphenolic compound isolated from cotton seeds, which are traditionally used in TCM for improving immunity. Gossypol is initially applied as a male antifertility agent. Following studies suggest its anticancer, antioxidant, antiviral and antiparasitic activities [107–109]. Gossypol preferentially acts on redox reactions catalyzed by NAD^+^/NADH-based enzymes such as LDH-A. Gossypol is a nonselective competitive LDH-A inhibitor and its anticancer activity appears to be associated with LDH-A inhibition [110, 111]. However, gossypol revealed significant toxicities including cardiac arrhythmias, renal failure, muscle weakness, and even paralysis, resulting in the stop of further development [112]. Galloflavin, a gallic acid derivative, was recently found to directly bind with LDH-A and LDH-B. Biological studies found that galloflavin could block aerobic glycolysis and trigger apoptosis in cancer cells without interfering cellular respiration. [113]. An antimalaria drug FX-11 was also reported to inhibit LDH-A activity and induce cancer growth arrest in both *in vitro* and* in vivo *experiments[114]. To explore active compounds from herbal medicine as LDH-A inhibitors, we investigated the effects of *Spatholobus suberectus, *a natural Chinese herb, on LDH-A activities in breast cancer cells. Our results showed that *Spatholobus suberectus *extractions significantly inhibited LDH-A activity in the breast cancer cells (unpublished data). Furthermore, we have conducted bioactivity-guided screening and epigallocatechin was identified as the main compound accounting for the herb anti-LDH-A function, the mechanism of which is correlated to accelerated HIF-1*α* proteasome degradation (unpublished data).

### 3.4. Others

**Figure 4:**
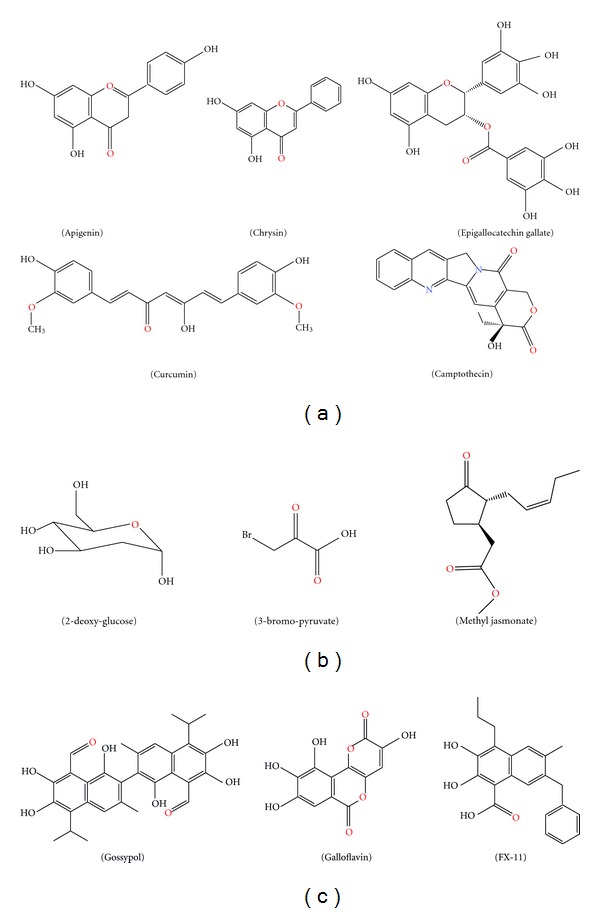
Chemical structures of glycolytic inhibitors derived from Chinese herbs. (a) Chemicals targeting on HIF-1*α*; (b) HKII inhibitors; (c) Chemicals targeting on LDH-A.

Glucose transporters (GLUTs) are important channels expressed on cell membrane for mediating glucose and other substrates entering into cells as nutrients. A total of six GLUT isoforms have been identified. Among them, GLUT-1 is closely related to cancer stages and chemo- or radiotherapy responses [115]. GLUT-1 silencing reduced cancer cell proliferation and mediated apoptosis [116]. For herb-derived inhibitors, apigenin, and genistein were proved to inhibit GLUT-1 [117, 118]. GAPDH is a classical glycolytic enzyme encoded by a “housekeeping gene” which is constitutively expressed in most cells. GAPDH is responsible for transforming glyceraldehydes-3-phosphate to 1,3-bisphosphoglycerate coupled with the reduction of NAD^+^ to NADH. Beside glycolytic function, GAPDH also participated in endocytosis, membrane fusion, vesicular secretory, nuclear tRNA transport, and DNA replication or repair. GAPDH inhibition resulted in induction of apoptosis [119]. Arsenic was demonstrated to abolish ATP generation in GAPDH-catalysing reaction process, although it is not in a direct binding mode [120]. AMP-activated protein kinase (AMPK) serves as a critical sensor in monitoring intracellular energy supply [121]. AMPK contributes to the increase of glycolytic activity in cancer cells. Thus, AMPK becomes a novel therapeutic target for cancer treatment. Herb-derived compounds curcumin and quercetin were demonstrated to induce apoptosis *via* AMPK pathway in cancer cells [122, 123]. In addition, other glycolytic enzymes including pyruvate kinase M2, glucose-6-phosphate isomerase, and transketolase-like enzyme 1, also participate in maintaining vitality of cancer cells. Development of small molecular inhibitors derived from herbs or natural plants targeting on these enzymes will be a new direction for anticancer research. The potential glycolysis inhibitors discussed above are summarized in the Figure 4.

 In summary, recent research progress indicate that many active compounds derived from herbal medicine have the potentials to regulate key metabolic enzymes, such as HIF-1*α*, GLUT-1, hexokinase, LDH-A, and PDK1. Those enzymes and proteins are important signaling molecules in the glycolytic pathways of cancer cells. The unique glycolytic pathways could provide cancer cells sufficient energy and ATP for their rapid proliferation and growth. In the meantime, the unique metabolic characteristics of cancer cells raise great opportunities for the development of anticancer agents targeting on aerobic glycolysis. With this strategy, the compounds derived from herbal medicine or synthesized novel chemicals would preferentially kill cancer cells instead of normal cells by blocking aerobic glycolysis, greatly facilitating the drug discovery for molecular target therapy.

## 4. Perspectives

The elucidation of specific molecular signaling involved in cancer initiation, development, and metastasis have provided the grounds for molecular targeting based therapeutic strategy. Glycolysis is an important hallmark of cancer cells differentiated from normal cells. The metabolic alternations and adaptations of cancer cells have been extensively studied in last decades. Tumor cells exhibit altered metabolic behavior due to tumor cell intrinsic properties and tumor microenvironment. With Warburg effect, tumor cells have increased glucose uptake and preferentially metabolize glucose through glycolysis even in the presence of oxygen, allowing them to sustain higher proliferation rates and fast growth. Therefore, targeting on glycolysis can be an important strategy in cancer prevention and treatment. For example, cancer cells acquire and develop resistance in many patients when receiving chemotherapy or radiotherapy. A recent study investigated the antitumor effects of trastuzumab (a monoclonal antibody against EGFR-2) in combination with glycolysis inhibitor 2-DG in ErbB2-positive breast cancer. Trastuzumab inhibited glycolysis *via* downregulation of heat shock factor 1 (HSF1) and LDH-A in ErbB2-positive cancer cells, resulting in tumor growth inhibition. Moreover, increased glycolysis *via* HSF1 and LDH-A contributed to trastuzumab resistance. Combining trastuzumab with glycolysis inhibition synergistically inhibited trastuzumab-sensitive and -resistant breast cancers *in vitro* and *in vivo*, due to more efficient inhibition of glycolysis [124]. Thus, inhibition of glycolysis may offer a promising strategy to overcome the resistances of cancer cells toward chemotherapy [125, 126]. Similarly, glycolysis based on Warburg effect also links to radioresistance [127]. Given many herb-derived phytochemicals exhibit properties of antiglycolysis and reversal of drug-resistance, Chinese herbal medicine targeting cancer glycolysis can be developed as an adjunct treatment for cancer patients by combining chemotherapy and radiotherapy. It will provide an opportunity to increase clinical outcome in cancer treatment.

There are 250,000 to 300,000 plant species in the world. Although large efforts are made, only 5,000 plant species have been studied for their possible medical applications. With the long history of application in human subjects, Chinese herbal medicine enjoys a unique position for molecular targeting-based therapeutic strategy. Based on histological documents and case reports in cancer treatment, Chinese herbal medicine provides a fast track and important source in drug discovery for molecular targeting based therapeutic strategy. It is anticipated that in the years to come, more and more medicinal herbs will be screened targeting on glycolytic-related molecular targets or other therapeutic targets. Establishing well validated high throughput screening platform is necessary and essential for the purpose, greatly accelerating the process of drug development. In conclusion, molecular-targeted screening strategy is critical and efficient strategy for exploring the active compounds from Chinese herbal medicine for anticancer drug discovery. It will not only bring the discoveries of new anticancer drugs with more target specific and low toxic, but also make contributions to the globalization and modernization of traditional Chinese medicine.

## Abbreviations

TCM: Traditional Chinese medicine
CAM: Complementary and alternative medicine
RCT: Randomized controlled trials
GLUT-1: Glucose transporter-1
SGLT-1: Sodium glucose cotransporter-1
PGK: Phosphoglycerate kinase
PK: Pyruvate kinase
LDH: Lactate dehydrogenase
MCT: Monocarboxylate transporter
GAPDH: Glyceraldehydes-3-phosphate dehydrogenase
PKM2: Pyruvate kinase, muscle
HIF-1: Hypoxia inducible factor-1
ODD: Oxygen-dependent degradation domain
VHL: von Hippel-Lindau
EGCG: Epigallacatechin gallate
HK: Hexokinase
VDAC: Voltage-dependent anion channel
2-DG: 2-Deoxy-glucose
3-BrpA: 3-Bromo-pyruvate
AMPK: AMP-activated protein kinase
HSF1: Heat shock factor 1.
